# Iron Chlorin e6 Improves Soybean Yield by Maintaining Chlorophyll Stability and Promoting Carbohydrate Accumulation Under Saline–Alkali Stress

**DOI:** 10.3390/plants15152411

**Published:** 2026-08-06

**Authors:** Wei Chen, Suyu Chen, Yanli Du, Liang Cao, Chunyuan Ren, Lu Lin, Xin Du, Jinghan Xu, Jiping Xu, Yuxian Zhang, Qiang Zhao

**Affiliations:** 1 Key Laboratory of Ministry of Agriculture and Rural Affairs of Soybean Mechanized Production, Heilongjiang Bayi Agricultural University, Daqing 163319, China; 2 Research Center of Saline and Alkali Land Improvement Engineering Technology in Heilongjiang Province, Daqing 163319, China; 3 National Coarse Cereals Engineering Research Center, Daqing 163319, China

**Keywords:** soybean, saline–alkali stress, Iron Chlorin e6 (ICe6), chlorophyll, transcriptomic analysis, metabolomic analysis

## Abstract

Saline–alkali stress is a widespread abiotic stress that severely impairs crop growth and yield formation. Iron Chlorin e6 (ICe6), a novel plant growth regulator, is essentially a chlorophyll derivative, and possesses potential application value in regulating plant chlorophyll metabolism and improving plant stress resistance. In this study, the saline–alkali-sensitive soybean cultivar Henong 95 and saline–alkali-tolerant soybean cultivar Hefeng 50 were used as experimental materials, and foliar spraying with 120 nmol/L ICe6 was conducted at the R1 stage. The results indicated that relative to CK, SA treatment markedly inhibited soybean growth, accompanied by reduced antioxidant capacity, excessive reactive oxygen species (ROS) accumulation and significantly lowered photosynthetic pigment content. Carbohydrate accumulation was substantially suppressed, which ultimately resulted in yield reduction (HN95: −12.31%; HF50: −11.08%). ICe6 treatment mitigated saline–alkali-induced growth inhibition in soybean plants, as reflected by markedly restored antioxidant indices, sharply decreased malondialdehyde (MDA), H_2_O_2_, and O_2_^−^ levels, and notably increased leaf area (HN95: +49.72%; HF50: +19.82%) and chlorophyll content (HN95: +36.06%; HF50: +90.75%). Combined transcriptomic and metabolomic profiling showed that, relative to the SA control, ICe6 treatment led to the identification of 2896 DEGs in HN95 and 3530 DEGs in HF50, with significant enrichment in photosynthesis- and chlorophyll metabolism-related pathways, e.g., GO:0009765 (photosynthesis, light harvesting). Differential metabolites were chiefly enriched in isoflavonoid biosynthesis—a source of antioxidants—and amino acid biosynthesis, which governs chlorophyll precursor synthesis. These findings suggest that ICe6 may enhance chlorophyll content by modulating the expression of genes involved in chlorophyll metabolism, contributing to light capture and chlorophyll biosynthesis, facilitating carbohydrate accumulation and ultimately contributing to increased yield under saline–alkali stress (HN95: +5.74%; HF50: +5.83%).

## 1. Introduction

Saline–alkali (SA) stress is a widely occurring abiotic stress in nature, which severely impairs plant morphogenesis and physiological processes [1]. Under saline–alkali stress, soybean plants exhibit stunted growth [2], leaf chlorosis, and reduced yield [3]. This is likely because saline–alkali stress induces the massive accumulation of reactive oxygen species (ROS) [4], triggers lipid peroxidation [5], and disrupts chloroplast structure, and ultimately impaired plant growth and final yield [6]. As the key pigment for plant photosynthesis, the content and stability of chlorophyll directly determine the photosynthetic efficiency, growth and development of plants [7,8]. Previous studies have shown that saline–alkali conditions interfere with the absorption and transport of iron and other elements essential for chlorophyll biosynthesis [9]. The uptake and translocation of these trace elements are critical during chlorophyll synthesis [10]. Reduced activity of chlorophyll biosynthetic enzymes and impaired iron absorption significantly inhibit chlorophyll biosynthesis in plants [11]. On the other hand, saline–alkali stress significantly downregulates the gene expression levels of key chlorophyll biosynthetic enzymes, including glutamyl-tRNA reductase (HEMA1), magnesium chelatase H subunit (CHLH), and protochlorophyllide oxidoreductase (POR) [12]. This inhibits the synthesis and accumulation of chlorophyll precursors, induces massive ROS accumulation during chlorophyll biosynthesis, damages chloroplast structure to accelerate chlorophyll degradation [13], and upregulates chlorophyllase activity to further promote chlorophyll breakdown. Collectively, these effects lead to decreased photosynthetic capacity, reduced carbohydrate accumulation, and impaired crop growth and final yield [14].

Studies have shown that ICe6 can alleviate combined saline–alkali stress in silage maize seedlings [15]. Other chlorophyll-related substances, such as 5-aminolevulinic acid (ALA), can significantly relieve the inhibition of salt stress on strawberry growth [16] and improve the adaptability of pepper seedlings to saline-alkali stress [17]. Hemin, another chlorophyll-related compound, has also been proven to mitigate NaCl stress by promoting photosynthesis and carbon metabolism in rice seedlings [18]. A plant-based modified biostimulant (copper chlorophyllin) has been confirmed to reduce oxidative stress induced by salt stress in Arabidopsis thaliana [19]. These studies collectively demonstrate that chlorophyll biosynthesis-related compounds can promote photosynthetic pigment synthesis under salt stress, alleviate leaf chlorosis and stunted growth caused by saline-alkali stress, and ultimately improve crop yield. Iron Chlorin e6 (ICe6), a chlorophyll derivative, is a novel and efficient plant growth regulator [20] that has been widely applied in crop physiological regulation and stress tolerance research, with remarkable progress in recent years. Hu et al. [21] reported that ICe6 promotes chlorophyll biosynthesis in wheat; Cao et al. [22] demonstrated that ICe6 alleviates the inhibitory effect of salt stress on rapeseed growth; Shao et al. [23] showed that ICe6 activates the antioxidant enzyme system in Andrographis paniculata, enhances ROS scavenging capacity, and mitigates oxidative damage caused by low temperature. However, the effects of ICe6 on chlorophyll metabolism and its underlying mechanisms in soybean under saline–alkali stress remain largely unknown.

In this study, two soybean cultivars, *Glycine max* cv. Henong 95 and *Glycine max* cv. Hefeng 50, were used as experimental materials, and four different treatments were set up. By analyzing the results of transcriptome sequencing and metabolome profiling after treatment with ICe6-soluble powder, combined with the effects of ICe6 on the morphogenesis, chlorophyll content, photosynthetic capacity, and antioxidant system of soybean plants under saline–alkali stress, we investigated the effects of ICe6 on chlorophyll metabolism-related pathways in soybean under saline–alkali stress and identified the key regulatory signaling pathways. This study provides a theoretical reference for the research on ICe6-mediated regulation of soybean response to saline–alkali stress.

## 2. Results

### 2.1. Effects of Iron Chlorin e6 on Plant Morphology and Biomass Accumulation of Soybean Under Saline–Alkali Stress

**Figure 1:**
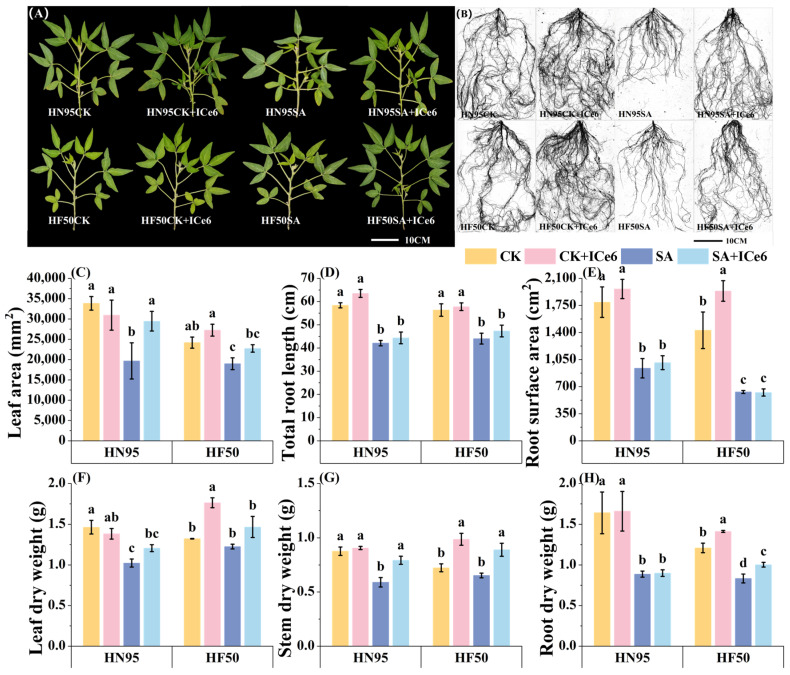
**Effects of ICe6 treatment on phenotypic characteristics and biomass of soybean plants under saline–alkali stress**, including shoot morphology (**A**), root morphology (**B**), leaf area (**C**), total root length (**D**), root surface area (**E**), leaf dry weight (**F**), stem dry weight (**G**), and root dry weight (**H**). Data are presented as the mean ± standard deviation (SD) of three independent experiments. Different letters indicate statistically significant differences among treatment groups (*p* < 0.05).

To investigate the effect of ICe6 on soybean growth under saline–alkali stress, morphological indicators, including leaf area, stem diameter, and dry weight of various tissues of HN95 and HF50, were measured under each treatment. As shown in Figure 1, compared with the CK treatment, SA treatment significantly reduced the leaf area of both soybean cultivars by 41.88% and 21.49% respectively. Moreover, root surface area decreased by 47.67% and 55.78%, and other morphological indicators such as leaf dry weight also declined significantly. Compared with the SA treatment, the leaf area of the two soybean cultivars under the SA + ICe6 treatment increased by 49.72% and 19.82%, and leaf dry weight increased by 17.92% and 19.56% respectively. Other morphological indicators including stem dry weight were improved to varying degrees. In addition, CK + ICe6 treatment also increased physiological indicators such as leaf area and tissue dry weight compared with CK, but the promoting effect was not significant. This may imply that ICe6 exerts a more obvious growth-promoting effect on soybean plants exposed to saline–alkali stress.

### 2.2. Effects of Iron Chlorin e6 on Antioxidant Capacity and Reactive Oxygen Species Accumulation in Soybean Under Saline–Alkali Stress

To investigate the effect of ICe6 on the antioxidant capacity of soybean under saline-alkali stress, NBT and DAB staining were performed on soybean leaves to observe the distribution of O_2_^−^ and H_2_O_2_, respectively. The contents of ROS and MDA, activities of antioxidant enzymes such as SOD, and contents of GSSG and GSH were also measured in HN95 and HF50 under each treatment.

**Figure 2:**
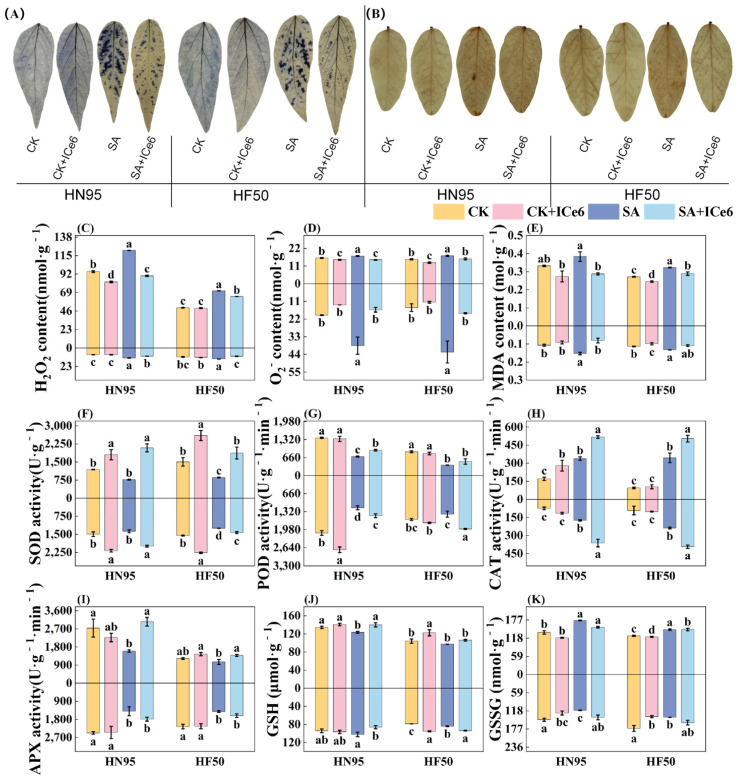
**Effects of ICe6 on the antioxidant defense system of soybean plants under saline**–**alkali stress**, including nitroblue tetrazolium (NBT) staining (**A**), 3,3′-diaminobenzidine (DAB) staining (**B**), hydrogen peroxide (H_2_O_2_) content (**C**), superoxide anion (O_2_^−^) content (**D**), malondialdehyde (MDA) content (**E**), superoxide dismutase (SOD) activity (**F**), peroxidase (POD) activity (**G**), catalase (CAT) activity (**H**), ascorbate peroxidase (APX) activity (**I**), reduced glutathione (GSH) content (**J**), and oxidized glutathione (GSSG) content (**K**). The upper half-axis indicates antioxidant parameters of leaves, and the lower half-axis represents antioxidant parameters of roots. Different letters indicate statistically significant differences among treatment groups (*p* < 0.05).

As shown in Figure 2A,B, compared with the CK treatment, SA treatment significantly increased the distribution of O_2_^−^ and H_2_O_2_ in soybean leaves. As shown in Figure 2C–E, compared with the CK treatment, SA treatment significantly increased the contents of H_2_O_2_, O_2_^−^ and MDA in both the leaves and roots of the two soybean cultivars. Compared with the SA treatment, SA + ICe6 treatment significantly reduced the contents of H_2_O_2_, O_2_^−^ and MDA in both leaves and roots. Notably, compared with CK treatment, the O_2_^−^ content in roots of the two soybean cultivars under SA treatment increased by 96.61% and 185.92% respectively, whereas these values decreased by 58.04% and 56.59% under SA + ICe6 treatment. The H_2_O_2_ content rose by 48.33% and 21.77% under SA treatment, and declined by 18.15% and 24.24% after SA + ICe6 application. Compared with the CK treatment, CK + ICe6 treatment also decreased the contents of H_2_O_2_, O_2_^−^ and MDA in both leaves and roots, but the differences were not statistically significant, which was consistent with the leaf staining results. This is possibly because ICe6 markedly modulates the antioxidant response of soybean plants only under stress conditions, whereas the scavenging effect of ICe6 on reactive oxygen species is negligible under normal growth conditions.

As shown in Figure 2F–I, compared with the SA treatment, SA + ICe6 treatment significantly increased the activities of SOD, POD and CAT in both leaves and roots, as well as APX activity in leaves of both cultivars, except for root APX activity. Compared with CK treatment, leaf POD activity of the two soybean cultivars decreased by 49.72% and 56.22% under SA treatment, and increased by 33.68% and 34.88% under SA + ICe6 treatment, respectively.

As shown in Figure 2J,K, compared with the CK treatment, SA treatment significantly increased oxidized glutathione content and decreased reduced glutathione content in both leaves and roots, resulting in a low GSH/GSSG ratio and leading to massive accumulation of reactive oxygen species (ROS), aggravating membrane lipid peroxidation and chlorophyll degradation. Compared with the SA treatment, SA + ICe6 treatment decreased oxidized glutathione content in leaves and restored the GSH/GSSG ratio to near-normal levels, restoring cellular redox homeostasis, alleviating damage to photosynthetic apparatus, and maintaining chlorophyll stability.

### 2.3. Effects of Iron Chlorin e6 on Transcriptome Profile of Soybean Under Saline–Alkali Stress

To analyze the mechanism by which ICe6 treatment enhances soybean tolerance to saline–alkali stress, comparative transcriptome analysis was performed on soybean leaves from SA and SA + ICe6 treatments of the two cultivars, with three independent biological replicates per sample. RT-qPCR was used to validate the transcriptome data, and the results showed that the transcriptome data were consistent with the RT-qPCR results (Supplementary Figure S1).

**Figure 3:**
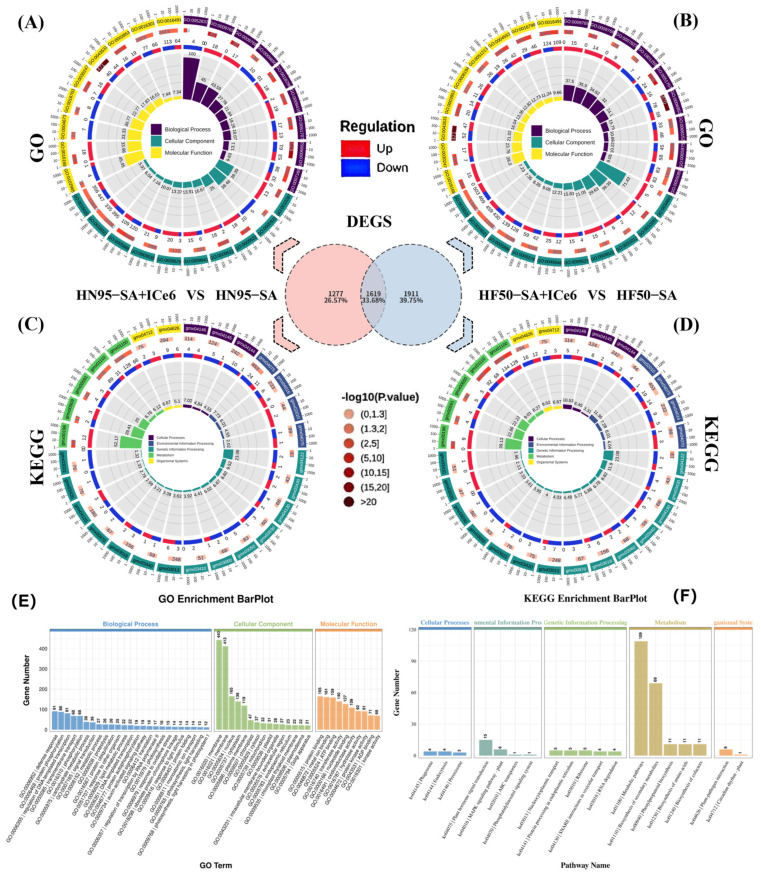
Transcriptomic GO enrichment analysis (**A**,**B**) and KEGG enrichment analysis (**C**,**D**) of two soybean cultivars before and after ICe6 application under saline–alkali stress; analysis of common differentially expressed genes (DEGs) in the transcriptome of two soybean cultivars before and after ICe6 application under saline–alkali stress (**E**,**F**), (|log_2_FC| ≥ 1 and *p* < 0.05).

As shown in Supplementary Figure S2, the Pearson correlation coefficients between biological replicates within each treatment group for HN95 were all higher than 0.99, indicating good sample reproducibility and reliable sequencing data. PCA analysis showed that PC1 (33.62%) and PC2 (24.28%) could effectively separate the samples from the two treatment groups. Differentially expressed genes (DEGs) were screened with the threshold of |log_2_(Fold Change)| ≥ 1 and FDR < 0.05. A total of 2896 DEGs were identified in HN95-SA + ICe6 vs. HN95-SA, including 1161 upregulated and 1735 downregulated genes. As shown in Figure 3A, GO enrichment analysis of DEGs in HN95 identified a total of 2046 enriched pathways, of which 230 were significantly enriched. As shown in Figure 3C, KEGG enrichment analysis identified a total of 119 enriched pathways, of which 19 were significantly enriched.

For HF50, the Pearson correlation coefficients between biological replicates within each treatment group were all higher than 0.991, indicating good sample reproducibility and reliable sequencing data. PCA analysis showed that PC1 (36.46%) and PC2 (20.32%) could effectively separate the samples from the two treatment groups. Using the same threshold, a total of 3530 DEGs were identified in HF50-SA + ICe6 vs. HF50-SA, including 1717 upregulated and 1813 downregulated genes. As shown in Figure 3B, a GO enrichment analysis of DEGs in HF50 identified a total of 2327 enriched pathways, of which 290 were noticeably enriched. As shown in Figure 3D, KEGG enrichment analysis identified a total of 123 enriched pathways, of which 18 were considerably enriched.

Compared with the SA treatment, the common DEGs of the two cultivars under the SA + ICe6 treatment were markedly enriched in 1536 GO terms, and their distribution is shown in Figure 3E. Among them, 153 GO pathways exhibit significant enrichment, and the pathways related to saline–alkali stress response mainly included 12 photosynthesis-related pathways, 10 lipid metabolism-related pathways, 10 signal transduction-related pathways, 14 cell structure and cytoskeleton-related pathways, 15 substance metabolism-related pathways, and 16 stress response and circadian rhythm-related pathways.

KEGG enrichment analysis of the common DEGs identified a total of 106 enriched pathways, of which 11 were significantly enriched, including photosynthesis—antenna proteins (ko00196), isoflavonoid biosynthesis (ko00943), betalain biosynthesis (ko00965), metabolic pathways (ko01100), and C5-branched dibasic acid metabolism (ko00660)—as shown in Figure 3F. These results suggest that compared with SA treatment, SA + ICe6 treatment may alleviate the inhibitory effect of saline–alkali stress on soybean growth by regulating the transcriptional levels of stress resistance systems and photosynthetic systems.

### 2.4. Effects of Iron Chlorin e6 on Metabolome Profile of Soybean Under Saline–Alkali Stress

**Figure 4:**
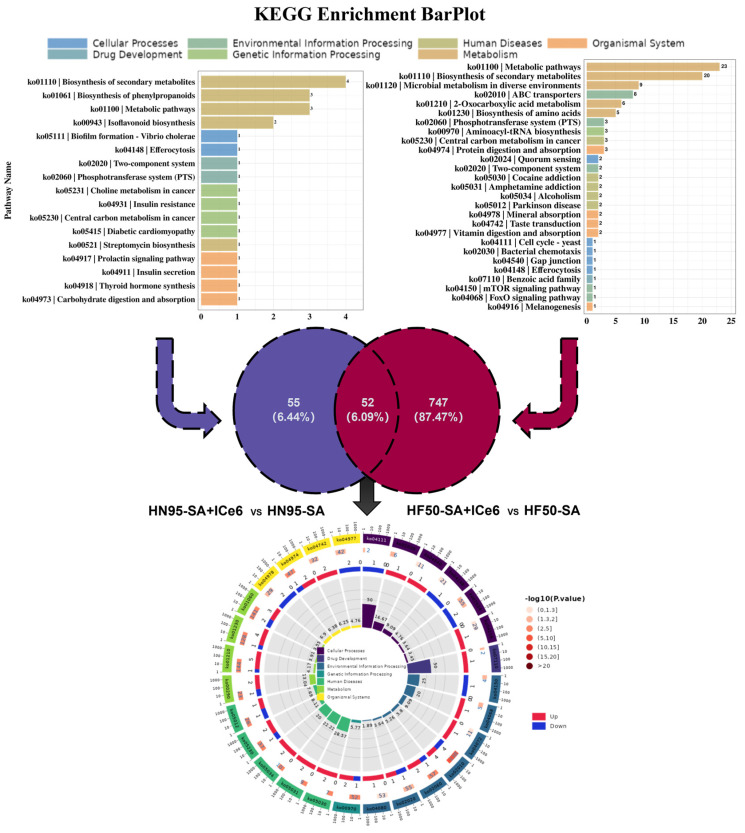
Metabolomic analysis of two soybean cultivars before and after ICe6 application under saline–alkali stress and the integrated metabolomic analysis of the two cultivars. (VIP > 1, ∣log_2_ FC∣ ≥ 1 and *p* < 0.05).

As shown in Figure 4, the metabolome changes in HF50 and HN95 before and after ICe6 spraying under saline–alkali conditions were compared, and the results of the two cultivars were comprehensively analyzed. All tissues were analyzed with six independent biological replicates.

As shown in Supplementary Figure S3, PLS-DA analysis of HN95 samples showed that Component 1 (39.29%) and Component 2 (28.84%) indicated good experimental reproducibility and reliable data. In HN95-SA + ICe6 vs. HN95-SA, a total of 107 differentially accumulated metabolites (DAMs) were identified, including 35 upregulated and 72 downregulated metabolites. KEGG enrichment analysis of the 35 upregulated DAMs in HN95 showed that they were concentrated in 26 pathways, of which 11 were markedly enriched, including biosynthesis of phenylpropanoids (ko01061), isoflavonoid biosynthesis (ko00943), efferocytosis (ko04148), streptomycin biosynthesis (ko00521), and carbohydrate digestion and absorption (ko04973).

For HF50, PLS-DA analysis showed that Component 1 (45.67%) and Component 2 (14.07%) indicated good experimental reproducibility and reliable data. In HF50-SA + ICe6 vs. HF50-SA, a total of 799 DAMs were identified, including 318 upregulated and 481 downregulated metabolites. KEGG enrichment analysis of the 318 upregulated DAMs in HF50 showed that they were concentrated in 129 pathways, of which 16 were significantly enriched, including ABC transporters (ko02010), valine, leucine and isoleucine biosynthesis (ko00290), biosynthesis of phenylpropanoids (ko01061), aminoacyl-tRNA biosynthesis (ko00970), and glyoxylate and dicarboxylate metabolism (ko00630).

A total of 52 common DAMs were identified between the two cultivars, and KEGG enrichment analysis showed that these DAMs were mainly concentrated in isoflavonoid biosynthesis (ko00943), biosynthesis of phenylpropanoids (ko01061), biosynthesis of secondary metabolites (ko01110), and metabolic pathways (ko01100). These results suggest that compared with SA treatment, SA + ICe6 treatment alleviates the inhibitory effect of saline–alkali stress on soybean growth mainly by regulating iron chelation and absorption, stress response, antioxidant defense, and energy metabolism.

### 2.5. Integrated Transcriptomic and Metabolomic Analysis of the Mechanism of Iron Chlorin e6 in Soybean Under Saline–Alkali Stress

**Figure 5:**
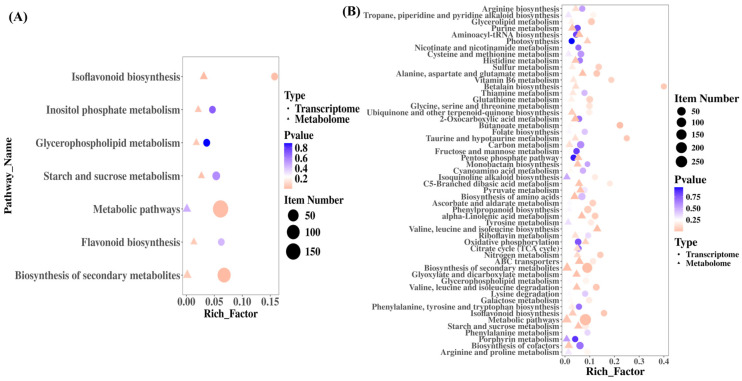
**Integrated transcriptomic and metabolomic analysis of two soybean cultivars before and after ICe6 application under saline**–**alkali stress** ((**A**): HN95-SA + ICe6 vs. HN95-SA; (**B**): HF50-SA + ICe6 vs. HF50-SA).

To clarify the relationship between gene expression and metabolite abundance, a correlation analysis and combined KEGG functional pathway analysis were performed using the significant DEGs and DAMs from HN95 and HF50 respectively. As shown in Figure 5A, in HN95, seven pathways were identified by combined transcriptomic and metabolomic analysis, among which the commonly enriched pathways were isoflavonoid biosynthesis (ko00943), biosynthesis of secondary metabolites (ko01110), and metabolic pathways (ko01100). As shown in Figure 5B, in HF50, 54 pathways were identified by combined analysis, among which the commonly enriched pathways were isoflavonoid biosynthesis (ko00943), alanine, aspartate and glutamate metabolism (ko00250), valine, leucine and isoleucine degradation (ko00280), metabolic pathways (ko01100), and biosynthesis of secondary metabolites (ko01110).

At the transcriptional level, the common DEGs of the two cultivars after SA + ICe6 treatment were significantly enriched in the photosynthesis—antenna proteins (ko00196) pathway and chlorophyll binding (GO:0016168). At the metabolic level, DAMs were significantly enriched in pathways such as amino acid biosynthesis (ko01230) and 2-oxocarboxylic acid metabolism (ko01210). Amino acid biosynthesis (especially the glutamate pathway) is the key precursor source for chlorophyll synthesis, while 2-oxocarboxylic acid metabolism provides the necessary carbon skeletons and energy supply for this process.

### 2.6. Effects of Iron Chlorin e6 on Chlorophyll Biosynthesis/Degradation Mechanisms and Chlorophyll Content in Soybean Under Saline–Alkali Stress

The above integrated transcriptomic and metabolomic analysis results provided a clear direction for subsequent focus on chlorophyll metabolism and photosynthetic function. The core mechanism by which ICe6 alleviates saline–alkali stress may lie in the positive regulation of chlorophyll metabolism and photosynthetic system function in soybean plants.

**Figure 6:**
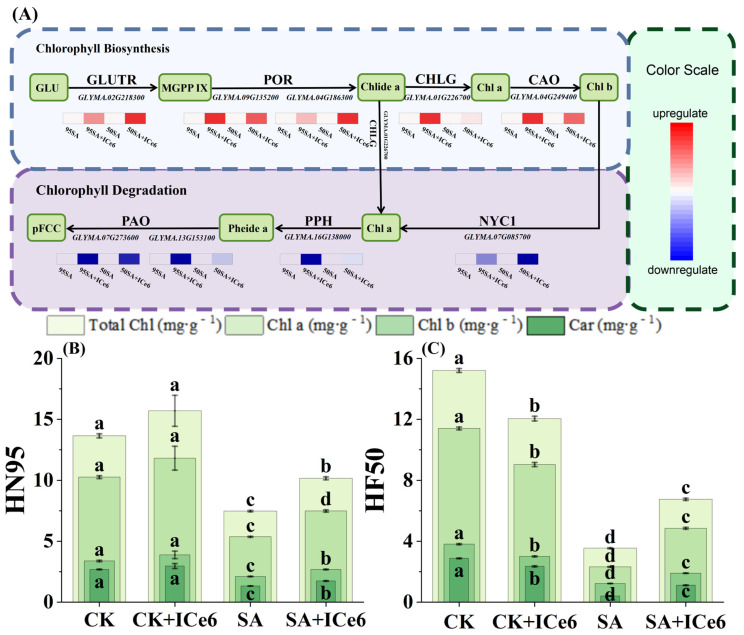
**Regulation of chlorophyll synthesis and degradation in soybean by ICe6 (relative expression of related genes under SA + ICe6 treatment was calculated with SA as the control)** (**A**). Chlorophyll content of soybean cultivar HN95 (**B**) and chlorophyll content of soybean cultivar HF50 (**C**). Different letters indicate statistically noticeable differences among treatment groups (*p* < 0.05).

As shown in Figure 6A, compared with the SA treatment, SA + ICe6 treatment upregulated the expression levels of chlorophyll biosynthesis-related genes in leaves of both HF50 and HN95, including *Glyma.02G218300* (encoding glutamyl-tRNA reductase, GLUTR/HEMA1), *Glyma.09G135200* and *Glyma.04G186300* (encoding protochlorophyllide oxidoreductase, POR), *Glyma.01G226700* (encoding chlorophyll synthase, CHLG), and *Glyma.04G249400* (encoding chlorophyll a oxygenase, CAO). In contrast, the expression levels of chlorophyll degradation-related genes were downregulated, including *Glyma.10G128400* (encoding HCAR), *Glyma.07G085700* (encoding NYC1), *Glyma.16G138000* (encoding pheophytinase, PPH), and *Glyma.13G153100* and *Glyma.07G273600* (encoding pheophorbide a oxygenase, PAO).

Meanwhile, the contents of chlorophyll a, chlorophyll b, carotenoids and total chlorophyll in leaves of HN95 and HF50 were determined. As shown in Figure 6B,C, compared with SA treatment, SA + ICe6 treatment significantly increased chlorophyll a content (39.45% and 109.63%), chlorophyll b content (27.37% and 55.32%), carotenoids content (31.24% and 77.5%) and total chlorophyll in both cultivars, and restored the light–energy conversion capacity of soybean leaves.

In summary, ICe6 can upregulate the expression levels of chlorophyll biosynthesis-related genes and downregulate the expression levels of chlorophyll degradation-related genes in leaves of both soybean cultivars under saline–alkali stress, ultimately promoting chlorophyll accumulation.

### 2.7. Effects of Iron Chlorin e6 on Gas Exchange Parameters and Chlorophyll Fluorescence Parameters of Soybean Under Saline–Alkali Stress

To investigate the effect of ICe6 on the leaf gas exchange parameters and chlorophyll fluorescence parameters of soybean under saline–alkali stress, net photosynthetic rate (Pn), stomatal conductance (Gs), intercellular CO_2_ concentration (Ci), transpiration rate (Tr), maximum photochemical efficiency of PSII (Fv/Fm), potential photochemical efficiency of PSII (Fv/Fo), actual photochemical efficiency of PSII (ΦPSII), and electron transport rate (ETR) were measured in leaves of HN95 and HF50.

**Figure 7:**
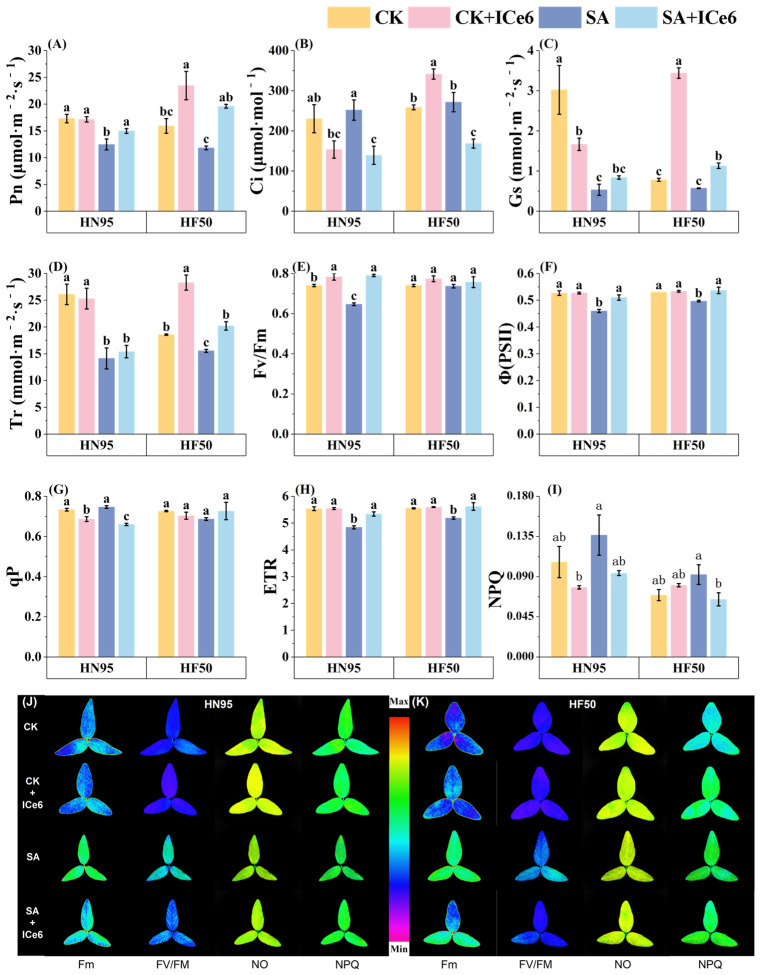
**Effects of ICe6 on photosynthetic capacity and chlorophyll fluorescence capacity of soybean plants under saline**–**alkali stress**, including net photosynthetic rate (**A**), intercellular CO_2_ concentration (**B**), stomatal conductance (**C**), transpiration rate (**D**), maximum photochemical efficiency of PSII (**E**), actual photochemical efficiency of PSII (**F**), photochemical quenching coefficient (**G**), electron transport rate (**H**), non-photochemical quenching (**I**), and chlorophyll fluorescence imaging (**J**,**K**). Different letters indicate statistically significant differences among treatment groups (*p* < 0.05).

As shown in Figure 7, compared with the CK treatment, SA treatment significantly reduced gas exchange parameters and chlorophyll fluorescence parameters in leaves of both HN95 and HF50. The net photosynthetic rate decreased by 27.95% and 25.74%, whereas the actual photochemical efficiency of photosystem II decreased by 12.66% and 6.28%, respectively, which severely impaired photosynthesis in soybean leaves. Compared with the SA treatment, SA + ICe6 treatment significantly increased the net photosynthetic rate of the two soybean cultivars by 20.20% and 65.82%, and elevated the actual photochemical efficiency of photosystem II by 10.87% and 8.05%, respectively. ICe6 alleviated the inhibitory effects of saline–alkali stress on photosynthetic and fluorescence parameters, and restored the light energy conversion capacity of soybean leaves.

### 2.8. Effects of Iron Chlorin e6 on Carbohydrate Content and Related Enzyme Activities in Soybean Under Saline–Alkali Stress

**Figure 8:**
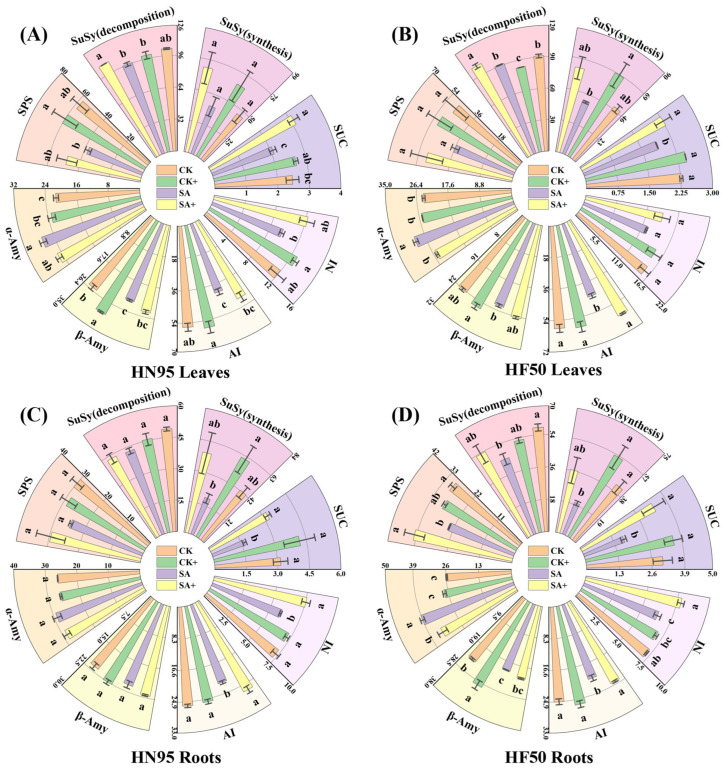
**Effects of ICe6 on carbon metabolism-related substances and enzyme activities in soybean plants under saline**–**alkali stress**. ((**A**): Carbon metabolism-related indicators in leaves of HN95; (**B**): Carbon metabolism-related indicators in leaves of HF50; (**C**): carbon metabolism-related indicators in roots of HN95; (**D**): carbon metabolism-related indicators in roots of HF50.) Different letters indicate statistically significant differences among treatment groups (*p* < 0.05).

As shown in Figure 8, compared with the CK treatment, leaf acid invertase activity of HN95 and HF50 decreased by 27.55% and 24.21%, neutral invertase activity reduced by 19.21% and 22.83%, and sucrose phosphate synthase activity dropped by 34.71% and 24.31% respectively. The reduction in acid invertase activity hinders sucrose decomposition, which ultimately results in insufficient grain filling. Declined neutral invertase activity leads to insufficient cellular energy supply. As a key enzyme involved in sucrose synthesis, reduced sucrose phosphate synthase activity seriously impairs normal carbohydrate accumulation in leaves and roots of soybean plants.

Compared with the SA treatment, SA + ICe6 treatment clearly increased acid invertase activity (19.56% and 38.14%), neutral invertase activity (26.62% and 16.47%), and sucrose phosphate synthase activity (28.57% and 37.5%) in the two soybean cultivars. The elevation of these enzyme activities alleviates the inhibitory effect of saline–alkali stress on carbohydrate accumulation in soybean. In addition, foliar application of ICe6 also increases the activities of carbon metabolism-related enzymes in leaves and roots of both cultivars under normal conditions, yet the promoting effect is more pronounced under saline–alkali stress.

### 2.9. Effects of Iron Chlorin e6 on Yield Traits of Soybean Under Saline–Alkali Stress

**Figure 9:**
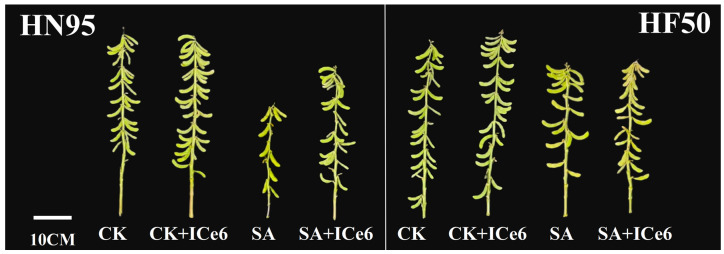
Effects of ICe6 on pod distribution of soybean plants under saline–alkali stress. Photographs were taken at soybean maturity stage.

As shown in Figure 9 and Table 1, compared with the CK treatment, SA treatment reduced pod number per plant by 31.52% and 27.42%, seed number per plant by 29.73% and 26.91%, 100-seed weight by 24.06% and 11.88%, and yield per plant by 12.35% and 11.16% in HN95 and HF50, respectively. Compared with SA treatment, SA + ICe6 treatment increased pod number per plant (23.01% and 17.05%) and seed number per plant (36.08% and 35.18%) of the two soybean cultivars, 100-seed weight (17.58% and 8.63%) and yield per plant (5.74% and 5.82%). Compared with CK, CK + ICe6 treatment slightly increased pod number per plant, seed number per plant and yield per plant; however, the increase in 100-seed weight was only significant in HF50. Collectively, SA + ICe6 mainly improved pod number per plant and seed number per plant of soybean. It is speculated that ICe6 may achieve yield enhancement by retaining more pods and seeds.

## 3. Discussion

Saline–alkali stress disrupts the physiological and metabolic balance of soybean plants [24] and inhibits the synthesis and transport of assimilates, thereby affecting organ development and biomass accumulation, and ultimately leading to yield reduction. Previous studies have shown that saline–alkali stress causes thinner stems, dwarf plants, and reduced nodule numbers in soybean [25]. Moreover, excessive ROS accumulation induced by saline–alkali stress leads to membrane lipid peroxidation, destroys normal cell structure and function, especially damaging the integrity of cell membranes [26], affects the function of various plant organs, and ultimately hinders normal plant growth.

ICe6 has antioxidant function, which can enhance the activity of plant antioxidant enzymes and promote ROS scavenging, and has potential application value in alleviating saline–alkali stress [27]. In this study, plant morphology and antioxidant-related indicators were measured. The results showed that saline–alkali stress markedly suppressed the growth of both soybean genotypes, accompanied by decreased antioxidant enzyme activities and excessive accumulation of ROS and MDA, which was consistent with the findings of previous studies [28]. Excessive accumulation of reactive oxygen species (ROS) may impair chlorophyll synthesis in plants, leading to leaf chlorosis. Foliar application of ICe6 significantly alleviated the growth inhibition and biomass reduction caused by saline–alkali stress. ICe6 treatment markedly improved the antioxidant enzyme activities of soybean plants, reduced ROS burst and membrane lipid peroxidation damage, and maintained cell membrane integrity. The stable functional status of plant organs provided sufficient material and energy support for soybean growth, thereby effectively relieving the inhibitory effect of saline–alkali stress on soybean development. To analyze the mechanism by which ICe6 alleviates soybean growth and development under saline–alkali stress, transcriptome and metabolome profiling were performed on HF50 and HN95 before and after ICe6 treatment under saline–alkali conditions. The results showed that compared with the SA treatment, the DEGs in the SA + ICe6 treatment were mainly concentrated in chlorophyll-related pathways such as chlorophyll binding (GO:0016168) and photosynthesis—antenna proteins (ko00196), which mainly included genes encoding key enzymes for chlorophyll synthesis and chlorophyll degradation.

As the core pigment for plant photosynthesis [29], chlorophyll content and light energy conversion efficiency directly determine plant energy acquisition, substance synthesis, growth and development, and ultimately yield [30]. Saline–alkali stress often promotes chlorophyll degradation [31], leading to decreased photosynthetic capacity, premature senescence, reduced crop quality [32,33], and lower yield, which is one of the key factors by which saline–alkali stress affects soybean growth.

As the core rate-limiting enzyme for chlorophyll degradation, pheophorbide a oxygenase (PAO) is localized on the inner membrane of chloroplasts and facilitates the oxidative breakdown of chlorophyll a. Under saline–alkali stress, PAO expression is generally upregulated, accelerating cell death. Transcriptomic data from the present study showed that ICe6 treatment downregulated the expression of pheophorbide a oxygenase (PAO), accompanied by concurrent chlorophyll accumulation in leaves, presenting consistent variation trends. Combined with previous reports demonstrating that reduced PAO activity delays chlorophyll degradation in rice [34], we speculate that ICe6 may participate in maintaining leaf chlorophyll homeostasis by regulating PAO expression. Nevertheless, functional validation of the PAO gene was not performed in this study, and the direct regulatory relationship between ICe6 and PAO requires further confirmation.

Metabolome KEGG enrichment analysis further revealed the response characteristics of ICe6 regulation to saline–alkali stress. Compared with the SA treatment, the DAMs in the SA + ICe6 treatment also showed significant pathway enrichment patterns, mainly concentrated in plant secondary metabolite biosynthesis (ko01060), phenylpropanoid biosynthesis (ko01061), isoflavonoid biosynthesis (ko00943), 2-oxocarboxylic acid metabolism (ko01210), and amino acid biosynthesis (ko01230).

Among them, the amino acid biosynthesis (ko01230) pathway directly determines the synthesis efficiency of chlorophyll precursors [35]. Chlorophyll biosynthesis starts with glutamate [36], and the activity of key enzymes such as glutamate synthase in this pathway directly regulates the synthesis and accumulation of glutamate in vivo [37]. Combined with pathways such as 2-oxocarboxylic acid metabolism (ko01210), they provide the necessary nitrogen skeletons and energy supply for chlorophyll biosynthesis [38,39]. Under saline–alkali stress, chlorophyll precursors are inhibited, leading to blocked chlorophyll synthesis, yellowing of soybean leaves, and reduced chlorophyll content. Flavonoids and isoflavonoids synthesized via the phenylpropanoid biosynthesis (ko01061) and isoflavonoid biosynthesis (ko00943) pathways have antioxidant functions in soybean plants [40,41], which can scavenge ROS produced under saline–alkali stress [42], reduce oxidative damage to chloroplast membrane structures, thereby indirectly maintaining chlorophyll homeostasis, further confirming the positive regulatory effect of ICe6 on chlorophyll synthesis. Although only 52 differential accumulated metabolites (DAMs) were shared between HN95 and HF50, accounting for approximately 49% of total DAMs in HN95. These 52 common DAMs were significantly enriched in three core pathways: isoflavonoid biosynthesis, phenylpropanoid metabolism, and amino acid precursor synthesis (chlorophyll precursor pathway). This finding supports the mechanism that ICe6 modulates chlorophyll homeostasis and enhances antioxidant capacity. As a salt-tolerant cultivar, HF50 possesses a more sophisticated metabolic network. Under saline–alkali stress, it can activate multiple auxiliary pathways including secondary metabolism and carbon/lipid metabolism to establish multiple compensatory salt-tolerance responses, resulting in a markedly larger number of DAMs. In contrast, the salt-sensitive cultivar HN95 exhibits weak metabolic regulation capacity. It only activates the conserved core stress-resistance pathways targeted by ICe6, while few significant alterations occur in other secondary metabolic pathways, leading to fewer cultivar-specific DAMs.

Integrated transcriptomic and metabolomic analysis further showed that the DEGs and DAMs of the two cultivars were commonly enriched in biosynthesis of secondary metabolites (ko01110), metabolic pathways (ko01100), starch and sucrose metabolism (ko00500), glycerophospholipid metabolism (ko00564), and isoflavonoid biosynthesis (ko00943) pathways. Among them, the isoflavonoid biosynthesis and secondary metabolite biosynthesis pathways can enhance plant antioxidant capacity [43,44], protect chloroplast structure and slow down chlorophyll degradation, thereby enhancing plant photosynthesis. Glycerophospholipid metabolism maintains chloroplast membrane stability [45], providing structural support for the photosynthetic system and chlorophyll. Starch and sucrose metabolism, as the core of photosynthetic carbon metabolism, regulates photosynthate accumulation and provides carbon skeletons for chlorophyll synthesis [46], providing material and energy support for its synthesis and ensuring normal photosynthesis. The above results are merely derived from integrated transcriptome and metabolome correlation analysis. Further experiments such as gene knockout are expected to verify the underlying molecular mechanisms.

The strength of photosynthesis often determines the carbon metabolism capacity of plants [47]. As the primary products of carbon metabolism, sucrose and starch can directly reflect the carbon metabolism level and photosynthate production capacity of plants [48]. The results of this study revealed that saline–alkali stress significantly suppressed gas exchange parameters and chlorophyll fluorescence parameters in both soybean cultivars, indicating that saline–alkali stress severely reduced the photosynthetic rate of soybean, leading to insufficient photosynthate production and hindering the accumulation of sucrose and starch. As an efficient plant growth regulator, ICe6 can alleviate the inhibitory effect of saline–alkali stress on carbon metabolism in soybean by activating the activities of metabolic enzymes. Measurements of carbohydrate contents and metabolic enzyme activities showed that ICe6 noticeably increased the activities of carbon metabolism-related enzymes in leaves and roots of the two soybean cultivars [28]. Specifically, increased activities of synthetic enzymes such as sucrose phosphate synthase and sucrose synthase promoted the synthesis of sucrose and starch, while moderate regulation of α-amylase and β-amylase activities reduced ineffective decomposition of carbohydrates and promoted efficient carbohydrate accumulation. These effects provided sufficient material and energy support for plant growth, ultimately increasing soybean yield under saline–alkali stress.

## 4. Materials and Methods

### 4.1. Materials, Growth, and Treatment Conditions

Two soybean cultivars, *Glycine max* cv. Henong 95 and Hefeng 50, were used as experimental materials in this study, which were provided by the Soybean Innovative Cultivation Team of the College of Agriculture, Heilongjiang Bayi Agricultural University. The test agent, 0.02% Iron Chlorin e6 (ICe6) soluble powder (Pesticide Registration No.: PD20190031), was supplied by Nanjing Baiter Biological Engineering Co., Ltd, Nanjing, China. Chernozem soil (pH = 7.0–7.3; available nitrogen: 44.31–54.00 mg·kg^−1^; available phosphorus: 11.15–13.65 mg·kg^−1^; available potassium: 138.14–178.00 mg·kg^−1^) was used for the normal growth conditions, while soda saline–alkali soil collected locally from Daqing, China (pH = 9.2–9.5; available nitrogen: 38.54–43.63 mg·kg^−1^; available phosphorus: 3.28–3.86 mg·kg^−1^; available potassium: 120.80–125.87 mg·kg^−1^) was used for the saline–alkali stress treatment.

Plump, intact-embryo, disease-free soybean seeds were sown in plastic pots (37 cm in height, 30 cm in upper diameter, and 27 cm in lower diameter, with six 1 cm diameter holes at the bottom) containing 25 kg of chernozem or soda saline–alkali soil, with a soil covering depth of 1.5 cm. At the true leaf stage, seedlings were thinned to uniform plants, with three plants retained per pot. Four treatments were applied at the R1 growth stage (beginning bloom): CK—soybean plants grown in chernozem soil + foliar spray of distilled water; CK + ICe6—soybean plants grown in chernozem soil + foliar spray of 120 nmol/L Iron Chlorin e6 (ICe6); SA—soybean plants grown in soda saline–alkali soil + foliar spray of distilled water; SA + ICe6—soybean plants grown in soda saline–alkali soil + foliar spray of 120 nmol/L Iron Chlorin e6 (ICe6).

The ICe6 concentration and application period used in this study were optimized through preliminary experiments. Samples were collected 7 days after treatment, frozen in liquid nitrogen, and stored at −80 °C for subsequent indicator measurements. Three plants per pot were considered one biological replicate. A total of eight pots were planted for each treatment. Among them, three pots were used for destructive sampling to determine physiological indicators, transcriptome, metabolome and other parameters; the remaining five pots were used for non-destructive measurements including phenotypic traits and chlorophyll fluorescence.

### 4.2. Measurement of Plant Morphology and Biomass Accumulation

Plant height was measured with a ruler, stem diameter was measured with a vernier caliper, and leaf area was determined using a leaf area meter (Yaxin-1241, Beijing Yaxin Liyi Technology Co., Ltd., Beijing, China). Root morphology was analyzed using a scanner (EPSON Scan 2.2) and the WinRHIZO Pro 2016a root scanning image analysis system (Regent Instruments, Inc., Quebec City, QC, Canada). Different tissues of soybean plants were separated, weighed for fresh weight, then dried at 105 °C for 30 min and adjusted to 85 °C until constant weight to determine dry weight. All measurements were performed with three biological replicates per treatment. At maturity, 10 plants were randomly selected from each treatment to measure pods per plant, seeds per plant, 100-seed weight, and yield per plant. All measurements were performed with three biological replicates per treatment.

### 4.3. Determination of Antioxidant-Related Indicators

The distribution of H_2_O_2_ was observed by DAB staining, and H_2_O_2_ content was determined by the potassium iodide method [49]. The distribution of O_2_^−^ was observed by NBT staining, and O_2_^−^ content was determined by the hydroxylamine hydrochloride method [49]. Malondialdehyde (MDA) content was measured by the thiobarbituric acid method [49]. Superoxide dismutase (SOD) activity was assayed by the nitroblue tetrazolium (NBT) method [49]. Peroxidase (POD) activity was determined by the guaiacol method [49]. The extraction method for ascorbate peroxidase (APX) and catalase (CAT) enzymes was the same as that for O_2_^−^ [49]. Root activity was measured by the triphenyl tetrazolium chloride (TTC) method. Electrical conductivity was determined by the boiling water method. Reduced glutathione (GSH) and oxidized glutathione (GSSG) contents were measured using commercial kits (G0206W48 and G0207F, Suzhou Grace Biotechnology Co., Ltd., Suzhou, China). All measurements were performed with three biological replicates per treatment.

### 4.4. Determination of Chlorophyll Content, Photosynthetic Gas Exchange Parameters and Chlorophyll Fluorescence Parameters

A total of 0.2 g of soybean leaf was soaked in 10 mL of 95% ethanol solution for 48 h. The absorbance values at 665 nm, 649 nm and 470 nm were measured with a spectrophotometer, and the contents of chlorophyll a, chlorophyll b, carotenoids and total chlorophyll were calculated according to the method of Yuan et al. [50]. Net photosynthetic rate (Pn), stomatal conductance (Gs), transpiration rate (Tr), and intercellular CO_2_ concentration (Ci) of the third fully expanded leaf from the top were measured 7 days after treatment using a LI-6400XT portable photosynthesis system (Li-COR Inc., Lincoln, NE, USA). Chlorophyll fluorescence parameters, including maximum photochemical efficiency of PSII (Fv/Fm), photochemical quenching coefficient (qp), actual photochemical efficiency of PSII (ΦPSII), apparent electron transport rate (ETR), and non-photochemical quenching coefficient (NPQ), were determined using a portable chlorophyll fluorometer (OS5P, OPTI-Sciences, Hudson, NH, USA). All measurements were performed with five biological replicates per treatment.

### 4.5. Determination of Carbohydrate Distribution and Related Enzyme Activities

The contents of soluble sugar, sucrose and starch in soybean leaves were determined according to the method of Du et al. [51]. Soluble sugar content was measured by the anthrone colorimetric method. Soluble protein content was determined by the Coomassie brilliant blue (G-250) staining method. The activities of sucrose metabolic enzymes, starch metabolic enzymes (α-amylase and β-amylase), sucrose phosphate synthase (SPS), sucrose synthase (SuSy), acid invertase and neutral invertase were assayed as described previously [52].

### 4.6. Transcriptome Profiling of Soybean Under Saline-Alkali Stress

Total RNA was isolated and purified using TRIzol reagent (Invitrogen, Carlsbad, CA, USA) following the manufacturer’s standard protocol. RNA concentration and purity were assessed using a NanoDrop ND-1000 spectrophotometer (NanoDrop, Wilmington, DE, USA), RNA integrity was verified with an Agilent 2100 Bioanalyzer (Agilent, Santa Clara, CA, USA), and agarose gel electrophoresis was performed for further confirmation. Only RNA samples meeting the following criteria were used for downstream experiments: concentration > 50 ng/μL, RNA integrity number (RIN) > 7.0, OD260/280 ratio > 1.8, and total RNA input > 1 μg [53]. Transcriptome sequencing and subsequent bioinformatic analysis were performed by LC Bio Technology Co., Ltd. (Hangzhou, China). Differential gene expression analysis was conducted using the DESeq2 software ((v1.32.0), with the screening thresholds of |log_2_FC| ≥ 1 and *p* < 0.05). Gene Ontology (GO) and Kyoto Encyclopedia of Genes and Genomes (KEGG) enrichment analyses of differentially expressed genes (DEGs) were performed using the DAVID online functional annotation tool (https://david.ncifcrf.gov/) (accessed on 19 November 2025) [54]. Enrichment analysis was performed using Fisher’s exact test, and the annotated genes of the soybean whole genome were adopted as the background gene set.

### 4.7. RT-qPCR Validation of Soybean Transcriptome Data Under Saline–Alkali Stress

RNA extraction and concentration determination were performed concurrently with transcriptome profiling. Single-stranded cDNA was synthesized using the Perfect Start Uni RT& qPCR Kit (TRAN, Beijing, China). RT-qPCR was performed on an ABI Step One™ Plus Real-Time PCR System using the Perfect Start Green qPCR Super Mix (TRAN, Beijing, China). *Tubulin A* was used as the internal reference gene [55,56]. The reaction procedure was as follows: initial denaturation at 94 °C for 30 s, followed by 40 cycles of 94 °C for 5 s, 60 °C for 15 s, and 72 °C for 15 s, with a final extension at 72 °C for 5 min. The relative expression levels of genes were calculated using the 2^−ΔΔCt^ method. A total of 23 genes were randomly selected for RT-qPCR verification. Each RT-qPCR experiment included three biological replicates and three technical replicates per sample [57,58]. The primer sequences used in this study are listed in Supplementary Table S1.

### 4.8. Metabolome Profiling of Soybean Under Saline–Alkali Stress

Metabolomic analysis was also performed by LC Bio Technology Co., Ltd. (Hangzhou, China). A total of 50 mg of soybean leaf tissue stored at −80 °C was ground and homogenized in 500 μL of ice-cold 80% methanol, followed by sonication. After centrifugation, the supernatant was collected, and the residue was re-extracted using the same procedure. The two supernatants were combined, and equal volumes of supernatant from all samples were pooled to prepare a quality control (QC) sample [59]. All metabolomic analyses were conducted with six biological replicates per treatment group.

Chromatographic separation was performed on a Waters ACQUITY UPLC HSS T3 column (100 mm × 2.1 mm, 1.8 μm) coupled with a Thermo Vanquish Flex ultra-high-performance liquid chromatograph and an Orbitrap Exploris 120 high-resolution mass spectrometer. Mobile phase A consisted of an aqueous solution containing 5 mmol/L ammonium acetate and 5 mmol/L acetic acid, and mobile phase B was acetonitrile. The elution gradient, flow rate and column temperature were set according to the standard protocol for the Lianchuan Orbitrap platform. Mass spectrometry data were acquired using electrospray ionization with alternating positive and negative ion modes.

Raw LC-MS data were subjected to peak extraction, alignment, normalization and metabolite annotation using XCMS and the metaX R package. Differential accumulated metabolites (DAMs) were screened based on three criteria: VIP **≥** 1 in the PLS-DA model, fold change (FC) > 1.2, and *p* < 0.05 from Student’s t-test. Gene Set Enrichment Analysis (GSEA) software (v4.1.0) was used for metabolite set enrichment analysis. KEGG pathways satisfying |NES| > 1, NOM *p* < 0.05 and FDR < 0.25 were defined as significantly enriched pathways between the two groups.

### 4.9. Data Analysis

Data were organized using Microsoft Excel 2016, analysis of variance (ANOVA) was performed using SPSS Statistics 21.0 software (Duncan’s multiple range test was used for multiple comparisons, with a uniform significance threshold set at *p* < 0.05 across all statistical analyses.), and graphs were plotted using OriginPro 2023 (64-bit).

## 5. Conclusions

This study revealed the mechanism of ICe6 on soybean growth under saline–alkali (SA) stress. Foliar application of 120 nmol/L ICe6 increased plant height, leaf area, total root length, and shoot/root biomass accumulation in both soybean varieties, alleviated SA-induced growth inhibition, reduced H_2_O_2_, O_2_^−^ and MDA contents, and elevated SOD, POD, CAT and APX activities in leaves and roots. ICe6 mitigated membrane lipid peroxidation, protected chlorophyll from degradation, upregulated chlorophyll synthesis-related genes and downregulated chlorophyll degradation-related genes, thereby increasing chlorophyll content, enhancing photosynthetic capacity and promoting carbohydrate accumulation. Ultimately, ICe6 improved pods per plant, 100-seed weight and yield per plant under SA stress. Notably, ICe6 showed a greater alleviating effect on soybean growth under SA stress than under normal conditions. These results provide a theoretical basis for the application of ICe6 in soybean salt–alkali tolerance improvement.

## Figures and Tables

**Table 1:** **Effects of ICe6 on soybean yield and its components under saline**–**alkali stress**. Different letters indicate statistically significant differences among treatment groups (*p* < 0.05).

Varieties	Treatments	Pods per Plant	Seeds per Plant	100-Seed Weight (g)	Yield per Plant (g)
HN95	CK	37 ± 1 a	71 ± 6 a	19.89 ± 0.72 a	12.51 ± 0.18 a
	CK + ICe6	34 ± 1 a	74 ± 5 a	19.49 ± 0.92 a	14.18 ± 0.55 a
	SA	25 ± 2 b	50 ± 3 b	15.11 ± 0.60 b	10.97 ± 0.24 b
	SA + ICe6	31 ± 3 ab	68 ± 3 a	17.76 ± 0.65 a	11.60 ± 0.27 a
HF50	CK	33 ± 1 a	74 ± 2 a	18.72 ± 0.35 a	13.02 ± 0.36 b
	CK + ICe6	30 ± 1 ab	76 ± 1 a	18.47 ± 0.22 a	14.79 ± 0.29 a
	SA	24 ± 4 b	54 ± 2 b	16.65 ± 0.72 b	11.48 ± 0.19 c
	SA + ICe6	28 ± 1 ab	73 ± 3 a	17.62 ± 0.21 ab	12.47 ± 0.58 bc

## Data Availability

The transcriptome data have been deposited in NCBI Gene Expression Omnibus (GEO) and are accessible through GEO Series accession number GSE334276 (https://www.ncbi.nlm.nih.gov/geo/query/acc.cgi?acc=GSE334276) (accessed on 4 June 2026). All metabolomic raw data have been deposited to the MetaboLights database under accession number MTBLS15147. Reviewers can access the dataset via the provided confidential reviewer link. https://www.ebi.ac.uk/metabolights/reviewerd6d1b54d-ff04-4cf6-9d1f-2808097a4139 (accessed on 24 July 2026); Access Token: mtbls_33b8b3e460e0c913356b03bd58d663f6aafc201ec3b4d3999d1c046a2c9abfa2.

## Supplementary Materials

The following supporting information can be downloaded at: https://www.mdpi.com/article/10.3390/plants15152411/s1, Table S1: Primer list; Figure S1: Correlation verification of RNA-seq and RT-qPCR expression levels for 23 random genes in HN95 and HF50. Figure S2: Transcriptome Correlation Analysis; Figure S3: Metabolome Correlation Analysis.
