# Supplementary material for: Iron Chlorin e6 Improves Soybean Yield by Maintaining Chlorophyll Stability and Promoting Carbohydrate Accumulation Under Saline–Alkali Stress
Source: Plants (Basel). 2026 Aug 6;15(15):2411. doi: 10.3390/plants15152411 (PMC13468497; doi:10.3390/plants15152411)
Supplement: Supplementary file 1 [file plants-15-02411-s001.zip › plants-4449720-supplementary.pdf]

plants

MDPI

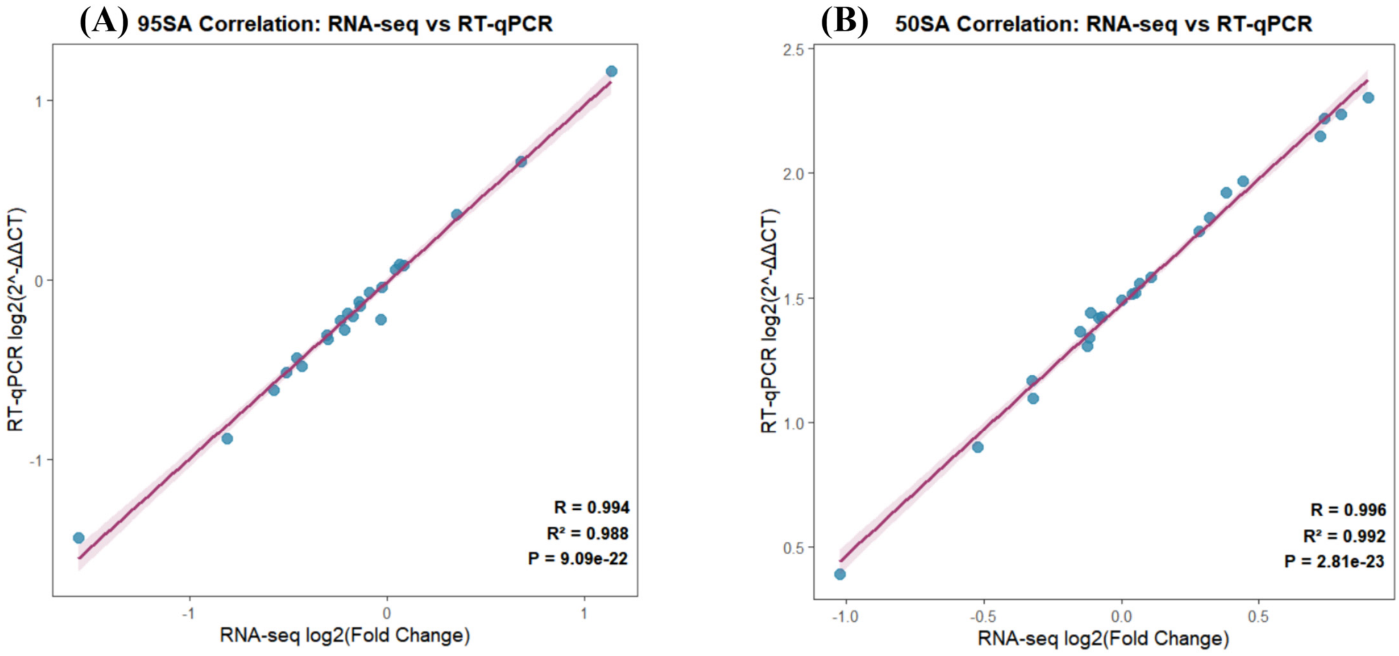

Figure S1. Correlation verification of RNA-seq and RT-qPCR expression levels for 23 random genes in HN95 (A); Correlation verification of RNA-seq and RT-qPCR expression levels for 23 random genes in HF50 (B). The X-axis represents $\log_2$(Fold Change) from RNA-seq, and the Y-axis represents $\log_2(2^{-\Delta\Delta CT})$ obtained by RT-qPCR.

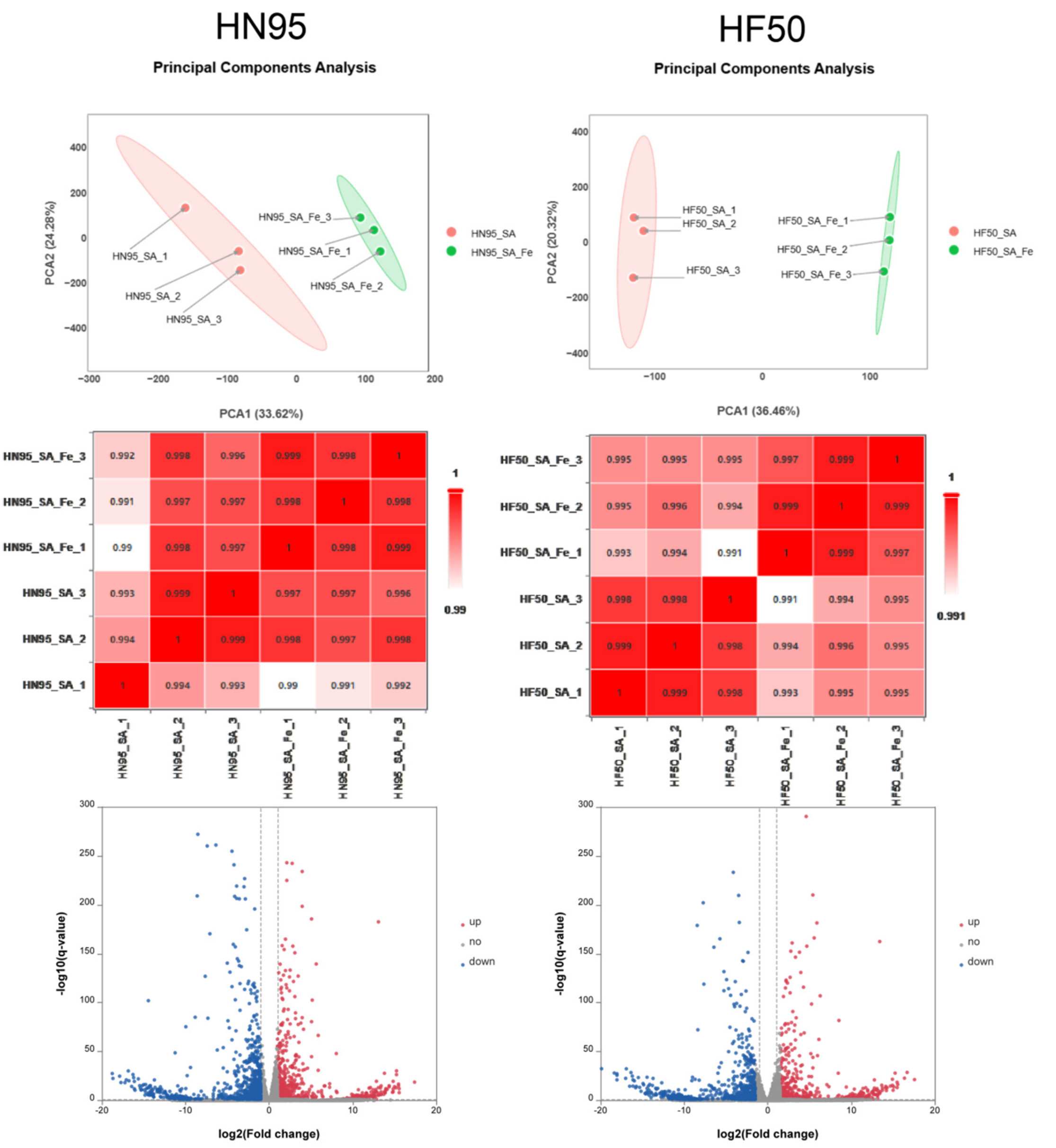

Figure S2. Transcriptome Correlation Analysis.

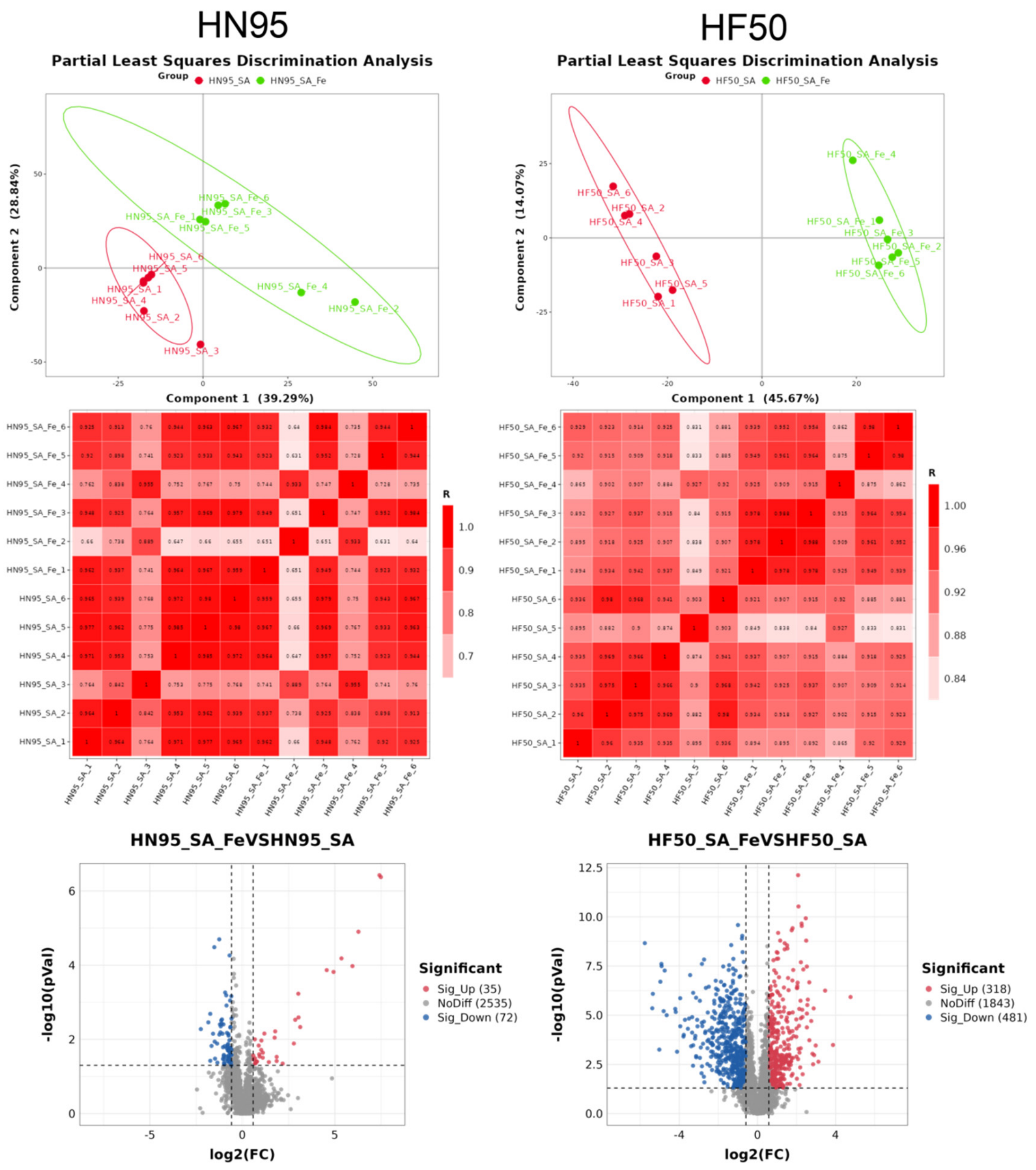

Figure S3. Metabolome Correlation Analysis.

Table S1. Primer list

Gene Name	Gene ID	Primer sequence (5'→3')
GMATM	GLYMA.04G182900-F	ACGTGCACTTCTCCGTGTCAAG
	GLYMA.04G182900-R	CCATGGATGCTTCGCATTTCGC
GMWEE1	GLYMA.03G211900-F	GCCTGGTCATTCGTTGCAACTTC
	GLYMA.03G211900-R	TTTAGCAGAAGGCCGCTTCACC
GMCDKA;1	GLYMA.09G030000-F	TTGTTAGGTTGCAGGATGTAGTGC
	GLYMA.09G030000-R	GCCGCGGATCTTTCACAAACTC
GMCYCB1;1	GLYMA.14G037100-F	CTGAGGAGCAACTAAGGGATTGCG
	GLYMA.14G037100-R	TGCCCTAAGCTTACTTCCTGGTG
GMRBR1	GLYMA.15G230300-F	ACCGTTTAAATGGCAACCGGAAG
	GLYMA.15G230300-R	TCAATGGTGCACCTGATGATGATG
GME2FA	GLYMA.02G198700-F	ATGACGGAAATGGTTCCTGCT
	GLYMA.02G198700-R	TCAGAGAATGCATGAGAAGAGAGC
GMHISTONE H4	GLYMA.18G064300-F	TGCCATGGATGTGGTTTATGCG
	GLYMA.18G064300-R	ACCAATGTCTTATCCTGCAACCC
GM-NBS1	GLYMA.14G208100-F	TGACTGTGGGAATGAGGACGTG
	GLYMA.14G208100-R	AGAACCAACAGTGCCTCGTGAAC
GMBRCA1	GLYMA.19G166900-F	CTGCATGCTTCCTCCATGTTGC
	GLYMA.19G166900-R	TAAGCAGCCCGACTTTGGCTTG
GMRAD51	GLYMA.13G115000-F	AGTAGATGGTTCTGCAGTCTTTGC
	GLYMA.13G115000-R	CTTTCCTGAGAGCTAGCCTCGTTG
GMMRE11	GLYMA.02G239200-F	TGGGCACTGACGAAAGCAAACC
	GLYMA.02G239200-R	GCATAACACAACCTGGCTGCAATG
GMKU70	GLYMA.20G248800-F	TCGAGGCGTTTATTGGGAGGAAAG
	GLYMA.20G248800-R	TTTCCGGTGAGATTGGCTGGTTG
GMKU80	GLYMA.05G125700-F	CTGGAATGTTTAGTTGCACTTCGC
	GLYMA.05G125700-R	ATCATTGAACTGTTCCGGTTCCTG
GMMLH1	GLYMA.04G254900-F	TGAAGAGAATACCTGTGATAATAC
	GLYMA.04G254900-R	TCCATCAGTAGCCATAGAAG
GMMSH2	GLYMA.17G121400-F	TCATCGGACGAAGGAGATATTACT
	GLYMA.17G121400-R	CACCCACCTGTCGGATAAATG
GMMSH6	GLYMA.19G216000-F	ATCCGAAGGTCTGTCTCT
	GLYMA.19G216000-R	AGCAACATTAACACCATAGC
GMMMH-2	GLYMA.13G366800-F	TGATCTGTTGGTCGGCTTGTGC
	GLYMA.13G366800-R	AGGCATCCTACTCTTGCCTTCC
GMOGG1	GLYMA.06G115500-F	TGGGTCCTAAAGTGGCTGCTTG
	GLYMA.06G115500-R	ACGTGTGTGTCAACAGGAACAGC
GMCSA1	GLYMA.04G045700-F	CGCTGATGAAATGGAAGGAACTGC
	GLYMA.04G045700-R	TGTCACACAAAGGGAGGCAAGAC
GMRAD4	GLYMA.05G166300-F	ATGGGAAGTGGCAACTGGAACC
	GLYMA.05G166300-R	ACTTGACCTCGCTCGTTCTTGG
GMNTH1	GLYMA.13G343700-F	GCAATCGTCTTGGATGGGTTTCG

	GLYMA.13G343700-R	GCAGCGCTTGTCTTGTTTCTTCC
GMNTH2	GLYMA.11G128600-F	TCAAGCCCATCTTCTTCCAAGTCC
	GLYMA.11G128600-R	TGTCCTTGTCCATGTCAAACCC
GMNTH3	GLYMA.04G031600-F	AGTCGCTCTTTACCATGCTCACC
	GLYMA.04G031600-R	AGAAGCTGAACTCCTTTCCTGACG
